# Genistein antagonizes gliadin-induced CFTR malfunction in models of celiac disease

**DOI:** 10.18632/aging.101888

**Published:** 2019-04-12

**Authors:** Speranza Esposito, Valeria Rachela Villella, Eleonora Ferrari, Romina Monzani, Antonella Tosco, Federica Rossin, Manuela D’Eletto, Alice Castaldo, Alessandro Luciani, Marco Silano, Gianni Bona, Gian Luigi Marseglia, Luigina Romani, Mauro Piacentini, Valeria Raia, Guido Kroemer, Luigi Maiuri

**Affiliations:** 1 European Institute for Research in Cystic Fibrosis, San Raffaele Scientific Institute, Milan 20132, Italy; 2 Department of Health Sciences, University of Eastern Piedmont, Novara 28100, Italy; 3 Regional Cystic Fibrosis Center, Pediatric Unit, Department of Translational Medical Sciences, Federico II University, Naples 80131, Italy; 4 Department of Biology University of Rome "Tor Vergata", Rome 00133, Italy; 5 Institute of Physiology, University of Zurich, Zurich 8057, Switzerland; 6 Department of Food Safety, Nutrition and Veterinary Public Health, Istituto Superiore di Sanità, Rome 00161, Italy; 7 Department of Pediatrics, Fondazione IRCCS Policlinico San Matteo, Pavia 27100, Italy; 8 Department of Experimental Medicine, University of Perugia, Perugia 06123, Italy; 9 National Institute for Infectious Diseases IRCCS “Lazzaro Spallanzani”, Rome 00149, Italy; 10 Equipe11 Labellisée Ligue Nationale Contrele Cancer, Centre de Recherche des Cordeliers, Paris 75006, France; 11 INSERM U1138, Centre de Recherche des Cordeliers, Paris 75006, France; 12 Université Paris Descartes/Paris V, Sorbonne Paris Cité, Paris 75006, France; 13 Metabolomics and Cell Biology Platforms, Institut Gustave Roussy, Villejuif 94805, France; 14 Pôle de Biologie, Hôpital Européen Georges Pompidou, AP-HP, Paris 75015, France; 15 Suzhou Institute for Systems Biology, Chinese Academy of Sciences, Suzhou 215123, China; 16 Karolinska Institute, Department of Women's and Children's Health, Karolinska University Hospital, Stockholm 17176, Sweden; * Equal contribution; # Senior co-authorship

**Keywords:** gluten peptides, celiac disease, inflammation, genistein, CFTR

## Abstract

In celiac disease (CD), an intolerance to dietary gluten/gliadin, antigenic gliadin peptides trigger an HLA-DQ2/DQ8-restricted adaptive Th1 immune response. Epithelial stress, induced by other non-antigenic gliadin peptides, is required for gliadin to become fully immunogenic. We found that cystic-fibrosis-transmembrane-conductance-regulator (CFTR) acts as membrane receptor for gliadin-derived peptide P31-43, as it binds to CFTR and impairs its channel function. P31-43-induced CFTR malfunction generates epithelial stress and intestinal inflammation. Maintaining CFTR in an active open conformation by the CFTR potentiators VX-770 (Ivacaftor) or Vrx-532, prevents P31-43 binding to CFTR and controls gliadin-induced manifestations. Here, we evaluated the possibility that the over-the-counter nutraceutical genistein, known to potentiate CFTR function, would allow to control gliadin-induced alterations. We demonstrated that pre-treatment with genistein prevented P31-43-induced CFTR malfunction and an epithelial stress response in Caco-2 cells. These effects were abrogated when the *CFTR* gene was knocked out by CRISP/Cas9 technology, indicating that genistein protects intestinal epithelial cells by potentiating CFTR function. Notably, genistein protected gliadin-sensitive mice from intestinal CFTR malfunction and gliadin-induced inflammation as it prevented gliadin-induced IFN-γ production by celiac peripheral-blood-mononuclear-cells (PBMC) cultured *ex-vivo* in the presence of P31-43-challenged Caco-2 cells. Our results indicate that natural compounds capable to increase CFTR channel gating might be used for the treatment of CD.

## Introduction

Celiac disease (CD), a food intolerance to dietary proteins from wheat, rye and barley, affects up to 1% of the world population [1–3]. In a subset of genetically susceptible individuals bearing the human leukocyte antigen (HLA) DQ2/DQ8, the ingestion of gluten triggers an immune reaction against gluten peptide, abolishing the normal state of oral tolerance and inducing an adaptive immune response against gluten-derived peptides with an autoimmune component [4–6]. This leads to the production of (diagnostic) autoantibodies against the self-antigen tissue transglutaminase 2 (TGM2) and eventually culminates in villous atrophy due to chronic intestinal inflammation [4–8]. In vivo, two peptides derived from the gluten component gliadin, a 33-mer (P56–88) and a 25-mer (P31–55), remain undigested [5,9,10]. P56–88, which contains the antigenic moiety of gliadin (the fragment P57-68), is deamidated by TGM2, binds to HLA-DQ2/DQ8, and induces an adaptive Th1 response. P31–55 is not recognized by T cells, but confers a potent adjuvant signal, in thus far that it induces an epithelial stress response with activation of TGM2 [5,9,10]. However, additional factors are required to perturb epithelial homeostasis and then to ignite the pathogenesis of CD. Such “external” triggers include reovirus infections [11] and perhaps other, yet-to-be-defined factors [8] that induce epithelial stress with TGM2 activation, upregulation of IL-15 and the cytotoxic activation of intraepithelial CD8^+^ T lymphocytes [5–8].

Recently, we discovered that the peptide P31-43, a fragment of P31–55, is able to trigger the epithelial stress response by binding to, and reducing the ATPase activity of, the nuclear binding domain 1 (NBD1) of the cystic fibrosis transmembrane conductance regulator (CFTR), thus inhibiting the chloride channel function of CFTR [12]. CFTR is a protein located at the surface membrane of epithelial cells (and other cell types) that does not only act as an anion channel but also functions as a hub protein that orchestrates multiple cellular signals [13–15]. Inherited loss-of-function mutations in the *CFTR* gene cause cystic fibrosis (CF), the most common life-threatening inherited disease in Caucasians [16,17]. In CF patients, impaired CFTR function fosters major pathogenic changes of the intracellular milieu including oxidative stress, TGM2 activation, autophagy impairment and alternated endosomal trafficking [15,18,19]. All these features are reminiscent of those induced by gliadin peptides in the intestine from CD patients. Indeed, the acquired CFTR dysfunction induced by P31-43 in the small intestinal mucosa provides the stress signal that alerts the innate immune response and ultimately enables the immune response against the antigenic moiety of gliadin [12].

CFTR oscillates between two distinct conformations, which reflect the open and closed states of the chloride channel [17,20]. P31-43 only binds to NBD1 when it is in the closed state, then blocking its gating function [12]. Stimulating CFTR channels by means of pharmacological “potentiators” such as VX-770 (Ivacaftor) or Vrx-532, which both increase the probability of CFTR channel opening [21,22], prevents P31-43 binding to CFTR, thus curtailing the pathogenic effects of P31-43 on the intestinal mucosa of gliadin-sensitive mice [12].

Several compounds that are authorized as over-the-counter food additives are endowed with the capacity to potentiate CFTR channel gating. Thus, genistein, a naturally occurring phytoestrogen contained in soy [23], is a widely recognized CFTR activator, acting both on isolated cells [24,25] and on tissues [26–28]. Driven by the consideration that natural compounds have a known safety profile facilitating their clinical implementation, we investigated the question as to whether genistein might be used to treat celiac disease. Here, we demonstrate that genistein is capable of preventing gliadin-induced CFTR malfunction and enteropathic effects in a variety of preclinical models.

## RESULTS

### Genistein prevents the pathogenic effects of P31-43 on intestinal epithelial cells

To determine whether genistein may protect intestinal epithelial cells from the gliadin-induced inhibition of CFTR function, we incubated confluent intestinal epithelial (Caco-2) cells, which are reportedly sensitive to gliadin or gliadin-derived peptides [10,29], for 3 to 24 h with the gliadin-derived peptide LGQQQPFPPQQPY (P31-43) (20 µg/ml) [9,10,12] in the presence or absence of a 20 min pre-incubation with genistein (50 µM). The peptide QLQPFPQPQLPY (P57-68) (20µg/ml) [9,10], which belongs to 33-mer sequence, as well as the scrambled peptide GAVAAVGVVAGA (PGAV) were used as controls [12].

**Figure 1:**
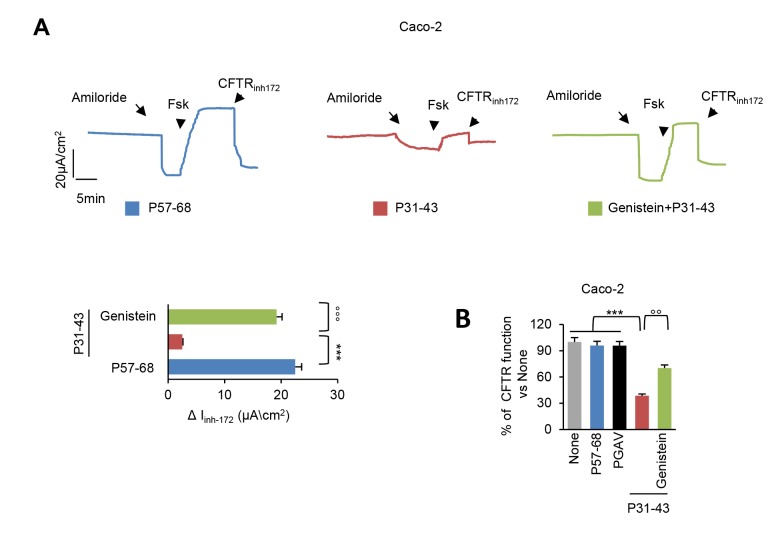
**Genistein prevents CFTR malfunction induced by P31-43.** (**A**) Representative traces of CFTR-dependent Cl- secretion measured by forskolin (Fsk)-inducible chloride current (Isc (μA/cm2)) in Caco-2 cells mounted in Ussing chambers after 3 h of incubation with P57-68 or P31-43 peptides (20 µg/ml), optionally after pre-treatment (20 min) with genistein (50 µM); quantification of the peak CFTR Inhibitor 172 (CFTRinh172)-sensitive Isc (∆Isc) in Caco-2 cells (n= 3 independent experiments). Means±SD of samples assayed; p***<0.001 P57-68 versus P31-43 challenge, °°°p<0.001 P31-43 versus genistein+P31-43 (ANOVA, Bonferroni post-hoc test). (**B**) Treatment of Caco-2 cells with P57-68, PGAV or P31-43 (3h) or with P31-43 after pre-treatment (20 min) with Genistein. Assessment of iodide efflux by SPQ fluorescent probe upon stimulation with forskolin (Fsk), expressed as percentage of CFTR function. Means±SD of samples assayed; ***p<0.001 P57-68 or PGAV challenge or untreated cells versus P31-43 challenge, °°p<0.01 P31-43 versus Genistein+P31-43 (ANOVA, Bonferroni post-hoc test).

Pre-incubation with genistein (50 µM) was effective in preventing the P31-43 induced decrease of the forskolin-inducible chloride current in confluent Caco-2 cells mounted in Ussing chambers (Figure 1A). The capacity of genistein to revert the inhibitory effect of P31-43 were comparable to those of VX-770 (Ivacaftor) or Vrx-532 [12]. The genistein-mediated reversion of P31-43 mediated CFTR inhibition was confirmed by means of other methodology based on measuring the rate of iodide efflux (Figure 1B).

**Figure 2:**
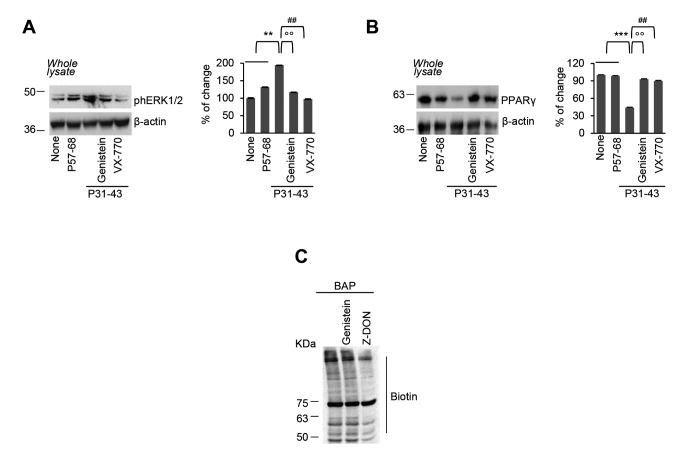
**Genistein prevents P31-43 induced epithelial stress response.** (**A-B**) Caco-2 cells were left untreated or incubated with P57-68 or with P31-43 in the presence or absence of VX-770 or genistein. Immunoblot of phospho-ERK 1/2 (**A**) or PPARγ (**B**) and densitometric analysis of protein levels relative to β-actin (*right*) (n=3 independent experiments). Means±SD of triplicates of independent experiments; **p<0.05 or ***p<0.001 untreated or P57-68 versus P31-43, °°p<0.01 P31-43 versus genistein+P31-43, ##p<0.01 P31-43 versus VX-770+P31-43 (ANOVA, Bonferroni post-hoc test). (**C**) In situ detection of TG2 activity, in Caco-2 cells pulsed with Ca^2+^, by immunoblotting of the TGM2-catalyzed incorporation of 5-biotinamidopentylamine (BAP) and blotting with anti-biotin antibody (*n*=3 independent experiments). Data information: The blots are representative of one experiment for group of treatment.

Next, we investigated whether pre-incubation with genistein (50 µM) would be capable of preventing the epithelial stress response and innate immunity activation induced by P31-43. Pre-incubation with genistein protected Caco-2 cells from signs of epithelial stress, as it prevented ERK 1/2 phosphorylation and PPARγ downregulation induced by P31-43 (Figure 2A-2B), as well as the reactive oxygen species (ROS) overproduction (SSupplementary Figure 1A). Notably, preincubation of Caco-2 cells with genistein controlled the P31-43 induced inflammation, secondary to TGM2 activation [12–14] (Figure 2A-2B), although genistein itself had no impact on TGM2-mediated transamidation reactions (Figure 2C).

**Figure 3:**
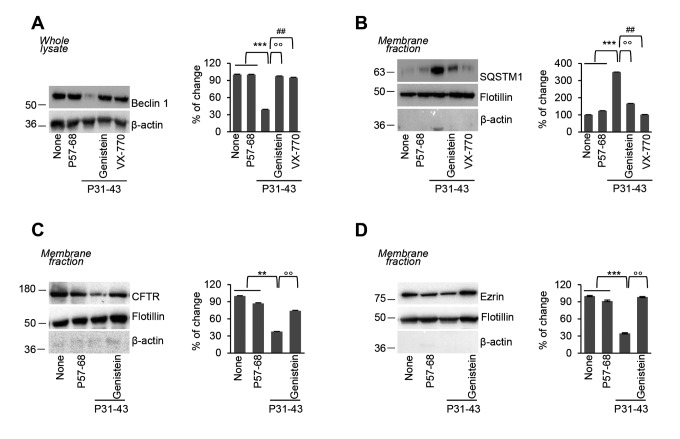
**Genistein prevents P31-43 induced Beclin 1 downregulation and plasma membrane CFTR disposal.** (**A-D**) Treatment of Caco-2 cells with P57-68 or P31-43 (3h) or with P31-43 after the optional pre-treatment (20 min) with genistein or VX-770. (**A**) Immunoblot of total lysate with anti-Beclin 1 antibody (*left*) and densitometric analysis of protein levels relative to β-actin (*right*) (n=3 independent experiments). Means±SD of triplicates of independent experiments; ***p<0.001 untreated or P57-68 challenged cells versus P31-43 treatment, °°p<0.01 P31-43 versus genistein+P31-43 or ##p<0.01 P31-43 versus VX-770+P31-43 (ANOVA, Bonferroni post-hoc test). (**B**) Immunoblot of membrane protein fractions with anti-SQSTM1 Ab and anti-flotillin as a control *(left)*, and relative densitometric analysis of immunoblot (*right*) (n=3 independent experiments). Means±SD of triplicates of independent experiments; ***p<0.001 untreated or P57-68 versus P31-43 treatment, °°p<0.01 P31-43 versus genistein+P31-43 or ##p<0.01 P31-43 versus VX-770+P31-43 (ANOVA, Bonferroni post-hoc test). (**C-D**) Immunoblot of membrane protein fractions with anti-CFTR (C) or Ezrin (D) and anti-flotillin as a control (*left*) and relative densitometric analysis of immunoblot (*right*) (n=3 independent experiments). Means±SD of triplicates of independent experiments; **p<0.01 or ***p<0.001 untreated or P57-68 challenged cells versus P31-43 treatment, °°p<0.01 P31-43 versus genistein+P31-43 (ANOVA, Bonferroni post-hoc test). Data information: The blots are representative of one experiment for group of treatment.

Interestingly, genistein prevented the decrease of Beclin 1 (BECN 1) protein levels, as well as the accumulation of the autophagic substrate sequestosome 1 (SQSTM1), that occurred upon P31-43 challenge (Figure 3A-3B). Conversely, the inactivation of autophagy by means of the pharmacological inhibitor 3-methyladenine (3MA) abolished the genistein-mediated protective effects on intestinal epithelial stress (as shown by the elevated PPARγ abundance and decreased ERK1/2 phosphorylation level; Supplementary Figure 2A-2B) triggered by P31-43, linking CFTR function, autophagy and maintenance of cellular homeostasis.

We previously demonstrated that the pharmacological [13] or P31-43 mediated [12] inhibition of CFTR function leads to CFTR disposal from the plasma membrane (PM) of epithelial cells owing to CHIP-mediated CFTR ubiquitination and subsequent SQSTM1 binding that diverts CFTR recycling to lysosomal degradation. In line with the protective effect of genistein against the P31-43 induced CFTR inhibition and accumulation of SQSTM1, pretreatment with genistein prevented the P31-43 induced disposal of mature CFTR from the plasma membrane (Figure 3C). Moreover, genistein negated the capacity of P31-43 to favour PM disposal of the CFTR interactor ezrin (Figure 3D), a key component of the ezrin–radixin–moesin (ERM) membrane complex that contributes to cytoskeleton organization and F-actin assembly.

**Figure 4:**
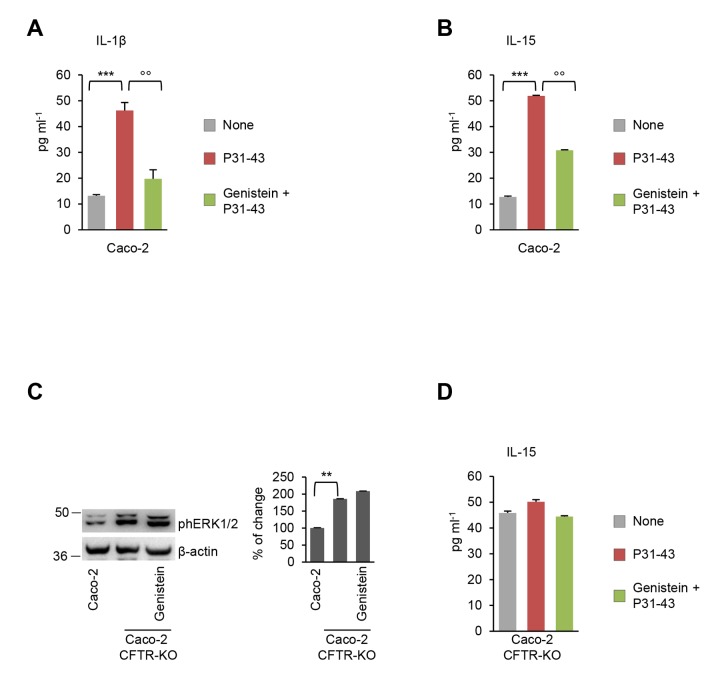
**Genistein opposes P31-43 induced inflammation in intestinal epithelial cells by targeting CFTR.** (**A-B**) Caco-2 cells incubated with or without P31-43 in the presence or absence of genistein. Protein levels (by specific ELISA) of IL-1 β (**A**) and IL-15 (**B**). Means±SD of pooled samples assayed in triplicates; ***p<0.001 untreated cells versus P31-43, °°p<0.01 P31-43 versus genistein+P31-43 (ANOVA, Bonferroni post-hoc test). (**C**) Immunoblot of phospho-ERK 1/2 in CFTR-WT Caco-2 cells or in CFTR-knock out Caco-2 cells treated or not with genistein (*left*) and densitometric analysis of protein levels relative to β-actin (*right*) (n=3 independent experiments). Means±SD of triplicates of independent experiments; **p<0.01 versus CFTR-knockout Caco-2 cells (ANOVA, Bonferroni post-hoc test). (**D**) CFTR-knockout Caco-2 cells incubated with P31-43 in the presence or absence of genistein. Protein levels (by specific ELISA) of IL-15. Means±SD of pooled samples assayed in triplicates. Data information: The blots are representative of one experiment for group of treatment.

We previously reported that P31-43 induces additional pro-inflammatory effects on Caco-2 cells, namely NLRP3 inflammasome activation and NF-κB p65 translocation into the nucleus with consequent increased IL-1β production and upregulation of IL-15 [12], a known mediator of the gliadin-induced enteropathy [5–8,30,31]. Importantly, genistein abolished all these P31-43 induced effects (Figure 4A-4B).

### Genistein prevents P31-43 induced inflammation by targeting CFTR

As it is true for most natural compounds, genistein is endowed with pleiotropic activities, including antioxidant and anti-inflammatory properties [32,33]. To determine whether the capacity of genistein to oppose P31-43 effects on epithelial cells is secondary to the potentiation of CFTR channel function, we used Caco-2 cells in which stable CFTR deletion was induced by CRISP/Cas9 technology (Caco-2_CFTR-KO_). In the absence of CFTR expression, genistein was unable to oppose the capacity of P31-43 to induce ERK1/2 phosphorylation (Figure 4C) and IL-15 upregulation (Figure 4D), thus confirming that genistein, similarly to VX-770 [12], controls the epithelial stress response and IL-15 production through on-target (via CFTR) rather than off-target effects.

Altogether, these results suggest that the genistein-mediated potentiation of CFTR channel activity might be taken advantage to treat the gliadin-induced enteropathy.

### Genistein prevents P31-43 induced manifestations in gliadin sensitive mice

**Figure 5:**
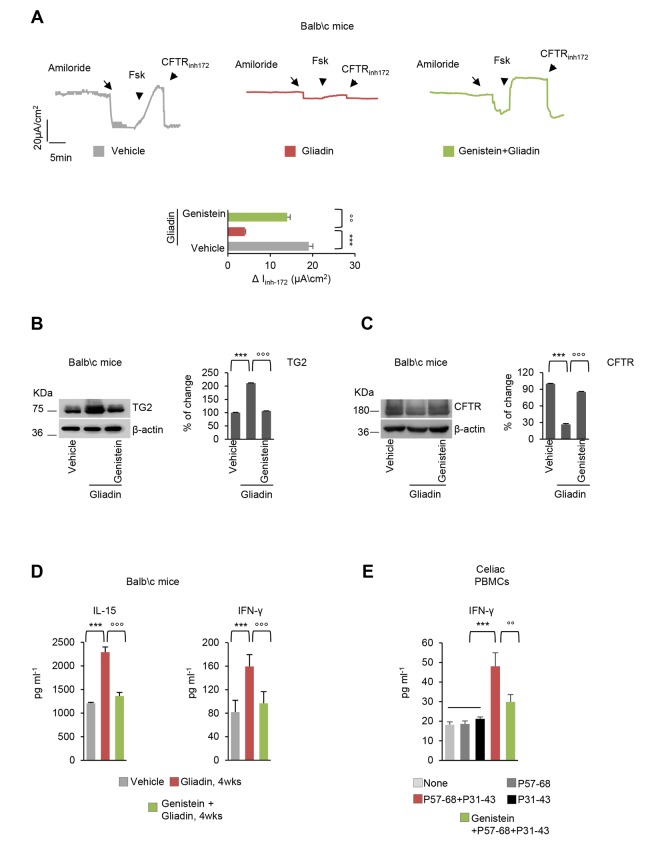
**Genistein protects gliadin-sensitive mice and celiac PBMC from the effects of gliadin.** (**A-D**) BALB/c mice fed with a gluten-free diet for at least 3 generations, orally challenged with vehicle or gliadin for 4 weeks (5 mg/daily for one week and then 5 mg/daily thrice a week for 3 weeks) in the presence or absence of intraperitoneal genistein administered 15 minutes prior gliadin challenge (*n*=10 mice per group of treatment). (**A**) Representative traces of CFTR-dependent Cl- secretion measured by forskolin (Fsk)- induced increase of chloride current (Isc (μA/cm2)) in small intestines mounted in Ussing chambers; quantification of the peak CFTR Inhibitor 172 (CFTRinh_172_)-sensitive Isc (∆Isc) in tissue samples (*n*=5-10). Means±SD of samples assayed; **p<0.001 gliadin versus vehicle, °°p<0.01 gliadin versus genistein+gliadin (ANOVA, Bonferroni post-hoc test). (**B-C**) Immunoblot with anti-TGM2 (**B**) or anti-CFTR (**C**) antibodies and β-actin loading control (*left*) and relative densitometric analysis of immunoblot (*right*) (*n*=3 independent experiments). Means±SD of triplicates of independent experiments; ***p<0.001 gliadin versus vehicle, °°°p<0.001 gliadin versus genistein+gliadin (ANOVA, Bonferroni post-hoc test). (**D**) Quantification of protein levels (by specific ELISA) of IFN-γ (*left*) and IL-15 (*right*). Means±SD of triplicates of independent pooled samples. ***p<0.001 vehicle vs gliadin or °°°p<0.001 gliadin vs genistein+gliadin (ANOVA, Bonferroni post hoc test). (**E**) IFN-γ release (ELISA) in culture supernatants by PBMC from 4 celiac patients cultured in the lower compartment of a bidimensional co-culture model upon 24 h challenge of confluent CaCo-2 cells in the upper compartment with P31-43 or P57-68 or with the combination of P31-43 and P57-68 in the presence or absence of genistein. Means±SD of triplicates of independent pooled samples. ***p<0.001, P57-68 or P31-43 vs P31-43/P57-68 combination (*n*=4); °°p<0.01, P57-68/P31-43 combination vs genistein+ P57-68/P31-43 (*n*=4), (ANOVA, Bonferroni post hoc test). Data information: The blots are representative of one experiment for group of treatment.

To investigate whether genistein may counteract the pathogenic effects of gliadin *in vivo* [34–39], we took advantage of an established mouse model of gliadin sensitivity. Three successive generations of BALB/c mice were fed with a gluten-free diet, and then young (10-week-old) mice were challenged with gliadin for 4 weeks (5 mg/daily for one week and then 5 mg/daily thrice a week for 3 weeks), following established protocols [12,34–37]. In this experimental model, genistein (25mg/kg in 100µl DMSO) was administered intraperitoneally 15 min prior to gliadin challenge. In all tested mice, genistein prevented the decrease of CFTR-dependent Cl^-^ secretion measured in small intestines mounted in Ussing chambers as the forskolin (Fsk)-inducible increase in chloride current (Isc (μA/cm2)) (Figure 5A). Beyond this electrophysiological effect, genistein prevented the upregulation of TGM2 (Figure 5B), as well CFTR disposal that is induced by gliadin challenge *in vivo* (Figure 5C). Moreover, genistein was effective in controlling the gliadin-induced IL-15 upregulation, as well as the increase of IFN-γ levels in mouse small intestines (Figure 5D).

In conclusion, our data indicate that genistein can reduce epithelial stress and local immune dysregulation induced by gliadin in vivo.

### Genistein opposes the gliadin-induced immune response ex vivo in celiac patients

To translate our findings into a relevant clinical setting, we determined whether genistein would prevent the HLA-restricted T cell activation induced by gliadin peptides. To this aim, we implemented a bidimensional co-culture model in which peripheral blood mononuclear cells (PBMC), collected from 4 celiac patients and 4 healthy controls, were placed in the lower compartment and confluent Caco-2 cells were placed in the upper compartment [40]. This experimental system aims at reproducing a mucosal environment in which the responsiveness of PBMC can be evaluated upon epithelial exposure to the relevant antigen. Confluent Caco-2 cells were challenged with a combination of P31-43 and P57-68 to elicit an immune response, as described [9–12], and IFN-γ was quantified in the supernatants from the lower compartment [40]. Genistein was effective in preventing the production of IFN-γ induced by the peptide combination (p<0.01) (Figure 5D).

These results suggest that genistein could represent an effective strategy to prevent or treat the gliadin-induced immunopathology.

## DISCUSSION

The inhibition of CFTR function perturbs cellular proteostasis as it causes two major alterations in cellular function, TGM2 activation and autophagy inhibition [12–15]. Autophagy is crucial for the adaptation to cell-autonomous and environmental triggers [14,15,41,42]. In this context, CFTR may be conceived as a major sensor of stress in thus far that it can activate autophagy when a stressful event risks to perturb cellular homeostasis [14,15,41]. Thus, CFTR, TGM2 and autophagy are engaged in a feed forward loop [13–15,43]. Indeed, CFTR dysfunction leads to TGM2 activation and consequent autophagy inhibition, while the restoration of autophagy and the inhibition of TGM2 re-establish CFTR function at the epithelial surface [13–15]. CFTR inhibition can result from CFTR mutations, as they are inherited by CF patients, or by acquired perturbation of CFTR channel function, as this occurs in CD [12–15,43]. Of note, the prevalence of CD is three-times higher in CF patients than in general population, meaning that CF predisposes to the development of CD [44–46]. In both conditions, an initially partial CFTR inhibition causes cells to derail in a spiral in which the activation of TGM2 and the inhibition of autophagy sustain an ever more severe inactivation of CFTR, thus locking cells in a condition of perturbed proteostasis and consequent chronic inflammation [43,47,48].

At present the therapy of CD is exclusively based on a gluten-free diet or the still experimental ingestion of enzymes that degrade pathogenic gliadin peptides within the gut lumen [49–51]. Other experimental strategies are emerging, such as tolerogenic vaccines to desensitize celiac individuals or strategies to prevent intestinal permeabilization [52–56]. In this perspective, targeting the initial pro-inflammatory reactions might represent an interesting option to prevent or treat CD. Our data suggest that this cascade of events might be interrupted at different levels within the “infernal trio” composed by CFTR inhibition, TGM2 activation and autophagy inactivation. At the apex synthetic drugs or natural compounds can be used to potentiate CFTR channel gating. Moreover, TGM2 inhibitors [12,15,18,19,29,57–59] or autophagy enhancers [60–64], can intercept the pro-inflammatory pathway downstream of CFTR malfunction.

We have previously demonstrated that Ivacaftor (VX-770), which is already used for the treatment of CF patients bearing plasma-resident CFTR mutants [65–69], can prevent P31-43 binding to CFTR, thus opposing the gliadin-induced CFTR malfunction with its pro-inflammatory consequences [12]. Here, we evaluated the ability of an established naturally-occurring CFTR potentiator, the over-the-counter nutraceutical genistein, to obtain similar effects. Apparently, genistein is as efficient as VX-770 in protecting the small intestine of gliadin-sensitive mice, human intestinal epithelial cells and PBMC from celiac patients against gliadin-induced inflammation.

Genistein is a biologically active isoflavone found in soy products [23]. As many naturally-derived compounds with beneficial effects on human health, genistein is endowed with multifaceted biological functions [25–28]. Beyond its ability to potentiate CFTR channel gating [25–28], genistein reportedly has a broad activity on a variety of diseases including cancer, insulin resistance, diabetes, obesity, chronic inflammation [32,33]. Characterizing the mechanisms through which over-the-counter nutraceuticals such as genistein may have a broad pro-health activity is crucial for defining appropriate indications and for designing discovery programs aimed at selecting more active compounds. Here, we demonstrate that genistein protects intestinal epithelial cells from the pro-inflammatory effects of P31-43 through an on-target effect, namely by stimulating CFTR function. Indeed, the protective effects of genistein against P31-43 induced epithelial stress were lost if CFTR was genetically removed from the system. It will be interesting to learn whether other natural compounds may have similar CFTR-stimulatory effects.

## MATERIALS AND METHODS

### Peptides

The following peptides were synthesized by Inbios (Napoli, Italy): α-gliadin peptide LGQQQPFPPQQPY (P31-43) or QLQPFPQPQLPY (P57-68) or scrambled GAVAAVGVVAGA (PGAV). All peptides were obtained with or without Biotin-NH2-tag.

### Cells and treatments

Human colon adenocarcinoma-derived Caco-2 and T84 cells were obtained from the ATCC. Cells were maintained in T25 flask in Modified Eagle Medium (MEM) for Caco-2, or Ham's F12 + DMEM (1:1) for T84, supplemented with 10% fetal bovine serum (FBS), 2mM Glutamine + 1% Non Essential Amino Acids (NEAA) and the antibiotics penicillin\streptomycin (100 units/ml) (all reagents from Lonza) [29]. Cells were grown in Transwells (Corning, 3470 or 3460) under the normal condition. Briefly, 8 × 10^4^ or 5 × 10^5^ cells were seeded in 6.5-mm diameter or 12-mm diameter, respectively, and grown until the RT reached 800 to 1,200 Ω·cm2. Transwells with a pore size of 0.4 μm were used. Medium in both the apical and basolateral chambers was changed every other day [40,58]. Cells were treated with 20 µg/ml of either α-gliadin peptide P31-43 or P57-68 or scrambled PGAV or modified P31-43 either biotin-tagged or not, for different time point (from 1h short challenge up to 24h) [12,70]. Caco-2 or T84 cells were also treated with: CFTR potentiators VX-770 (10μM) or Genistein (50μM) (Sigma Aldrich), TG2 inhibitor Z-DON (20nM, Zedira), and with autophagy inhibitor 3-methyladenine (3-MA, 3mM, Sigma Aldrich)

### Mice and treatments

BALB/c mice (background BALB/cAnNCrl) [35] were purchased from Charles River (Varese, Italy). Three-generation gluten-free diet (Mucedola srl, Milan), male and female, were challenged with gliadin for 4 weeks [34–37]. To assess the effects of Genistein or VX-770 into a controlled environment, mice were challenged via gavage for 4 weeks with i) vehicle alone or ii) gliadin (Sigma-Aldich, G3375) (5 mg/daily for one week and then 5 mg/daily thrice a week for 3 weeks) [12,34–37] in the presence or absence of intraperitoneal genistein (25mg\kg in 100µl DMSO, Sigma-Aldrich) administered 15 minutes prior each gliadin challenge (n=10 mice per group of treatment).

At the end of the last daily treatment, mice were anesthetized with Avertine (tribromoethanol, 250 mg/kg, Sigma Aldrich, T48402) and then killed; the intestines were collected for CFTR function analysis or stored for all described techniques.

These studies and procedures were approved by the local Ethics Committee for Animal Welfare (IACUC No849) and conformed to the European Community regulations for animal use in research (2010/63 UE).

### Purification of PBMC and transwell co-culture model

Five ml of peripheral blood have been withdrawn from 4 untreated celiac patients (females and males, age range 8-25 years) and from 4 not CD-affected controls. The Ethics Committee of the Istituto Superiore di Sanità (ISS) approved the protocol (#CE/12/341), and patients or patients’ parents signed the informed consent. Peripheral blood mononuclear cells were isolated using lympholite (Cederlane, UK) density gradient overlaid by heparin blood diluted 1:1 in PBS and centrifuged (20 min at 900 rpm). After being washed three times, PBMCs were resuspended in complete RPMI 1640 supplemented with 25 mM HEPES, 10% (v/v) heat-inactivated FBS, 100U/ml penicillin, 100 mg/ml streptomycin, and 1% 2 mM l-glutamine.

For transwell experiments using polarized Caco-2 cells, 3 weeks prior to the experiment, Caco-2 cells were seeded at a density of 80 × 10^3^ cells x cm^2^ on 0.4-μm, 1-cm^2^ tissue culture inserts (Costar, Corning Incorporated). Transwell cultures (12-well) with confluent Caco-2 monolayers were used for co-culture with 1 ml PBMC (1.5 × 10^6^ cells/ml) using PBMC medium and kept in an incubator at 37 °C and 5% CO2. Cells were allowed to settle for 1 h before the starting of the experiment. The permeability of the epithelial monolayer was assessed just before the experiments, measuring the transwell electrical resistance between the upper and lower compartments. A value of transwell resistance >800 Ω × cm2 has been considered index of a fully formed epithelial monolayer, not allowing the paracellular passage of molecules [40].

Caco-2 cells were apically exposed for 3 h with P31-43 peptide (20 μg/ml) and then treated with P57-68 (20 μg/ml) in presence or absence of CFTR potentiators Genistein. As negative control, cells were treated with medium alone, and with P57-68 alone. After the treatments, supernatants from the basolateral compartment were collected, centrifuged, and stored at -20°C until cytokine measurement. At the same time, the cells from the apical compartment were harvested, lysated, and stored at −80 °C.

### Ussing chamber

Chambers for mounting either transwell cell cultures or mouse tissue biopsies were obtained from Physiologic Instruments (model P2300, San Diego, CA, USA). Chamber solution was buffered by bubbling with identical Ringer solution on both sides and were maintained at 37°C, vigorously stirred, and gassed with 95%O2/5% CO2. Cells or tissues were short circuited using Ag/AgCl agar electrodes. A basolateral-to-apical chloride gradient was established by replacing NaCl with Na-gluconate in the apical (luminal) compartment to create a driving force for CFTR-dependent Cl^−^ secretion. To measure stimulated Isc, the changed sodium gluconate solution, after stabilization, was supplied with 100µM amiloride. Agonists (forskolin) were added to the bathing solutions as indicated (for a minimum 5 min of observation under each condition) to activate CFTR channels present at the apical surface of the epithelium (either cell surface or lumen side of the tissue) and CFTR_Inh-172_ (10µM) was added to the mucosal bathing solution to block CFTR-dependent Isc. Short-circuit current (expressed as Isc (μA/cm2)) and resistance were acquired or calculated using the VCC-600 transepithelial clamp from Physiologic Instruments and the Acquire&Analyze2∙3 software for data acquisition (Physiologic Instruments), as previously described [12,71–74].

### CRISP/Cas9 CFTR knockout

CFTR CRISP/Cas9 KO plasmids were purchased from Santa Cruz Biotechnology and transfected in Caco-2 cells by UltraCruz trasfection reagent according to the manufacturer's instructions (Santa Cruz Biotech.). Successful transfection of CRISPR/Cas9 KO Plasmid was visually confirmed by detection of the green fluorescent protein (GFP) by immunofluorescece. The cells were then sorted by replacing selective media with Puromycin antibiotic approximately every 2–3 days for a minimum of 3–5 days. The knockout was then confirmed by western blot with specific CFTR antibody and by functional assay (Ussing chamber or SPQ assay) [12].

### Immunoblot

The whole lysate or membrane fraction proteins of cell lines and mice intestine homogenates were obtained from treated and untreated cells or mice as described [12,13,15,18,19,58,75–79]. The equal amount of protein were resolved by SDS-PAGE gel and blotted with antibodies against: SQSTM1, (Sigma Aldrich, 108k4767)1:1000, PPARγ (Santa Cruz Biotechnology, sc7273) 1:500, BECN1 (Abcam, ab58878) 1:1000, CFTR clone M3A7 (Abcam, ab4067) 1:500, phospho- ERK1/2 (php42/44, Cell Signaling Technology, #91101) 1:1000, Ezrin (BD, 610603) 1:1000, biotin (Abcam, ab1227 )1:2000, TG2( CUB Novus Bio) 1:1000 used as primary antibodies. Normalization was performed by probing the membrane with anti-β-actin (Cell Signaling, #4970) 1:1000, and anti-flotillin (Abcam ab15148) 1:1000 antibodies.

### Membrane fractionation

Protein from membrane fractionation were obtained as described [13,15,18,19,71,76]. Cells were homogenized with a Potter-Elvehjem pestle and centrifuged at 2300xg for 15 min at 4 °C. Supernatants that contains the cytoplasmic and PM fractions were centrifuged 1 h at 16000 xg at 4 °C; the pellet was the intact membrane and was solubilized in BUFFER A (20mM Tris-HCl pH 7.4, 2mM EDTA, 20mM 2-ME, 1X PMSF, 1 µg/ml inhibitor protease cocktail) +1% Triton X-100 and centrifuged 1 h at 60000 xg in the ultracentrifuge. The supernatants were collected as PM fraction. Proteins of PM fraction were used for WB and immunoblotted against CFTR, Ezrin, SQSTM1\p62 and flotillin antibodies.

### ELISA

ELISA analysis was performed on tissue samples using standard ELISA kits (R&D Systems) for IL-1β, IL-15, INF-γ. According to the manufacturer’s instructions. Samples were read in triplicates at 450 nm in a Microplate Reader (BioRad, Milan, Italy) using Microplate Manager 5.2.1 software. Values were normalized to protein concentration evaluated by Bradford analysis.

### TGM2 enzyme activity detection

TG2 enzymatic activity in Caco-2 cells, treated as described above, was detected by incorporation of 5-(biotinamido)pentylamines (BAP) into protein substrates. For BAP-incorporation, 2 mM BAP (Soltec Ventures, B110) were directly added into the medium together with the indicated treatments. In the presence of TG2 transamidating activity, BAP is incorporated into the substrates. To measure this activity, cells were lysed and proteins were resolved by SDS-polyacrylamide gel. The blots were incubated with anti-Biotin antibody [80,81].

### ROS detection

The cells were pulsed with 5μM CellROX Green Reagent (C10444, Thermo Fisher Scientific) for 10 min in live-cell imaging at 37 °C. After washing, the cells were subsequently analysed by confocal microscopy [82].

### Statistical analysis

GraphPad Prism software 6.01 (GraphPad Software) was used for analysis. Data were expressed as means±SD. Statistical significance was calculated by ANOVA (Bonferroni's post hoc test) for multiple comparisons and by Student's t-test for single comparisons. We considered all P values 0.05 to be significant. The in vivo groups consisted of ten mice/group. The data reported are representative of at least three experiments.

## Supplementary Material

Supplementary Figures
