# Supplementary material for: Genistein antagonizes gliadin-induced CFTR malfunction in models of celiac disease
Source: Aging (Albany NY). 2019 Apr 12;11(7):2003–19. doi: 10.18632/aging.101888 (PMC6503870; doi:10.18632/aging.101888)
Supplement: Supplementary Figures [file aging-11-101888-s001.pdf]

SUPPLEMENTARY FIGURES

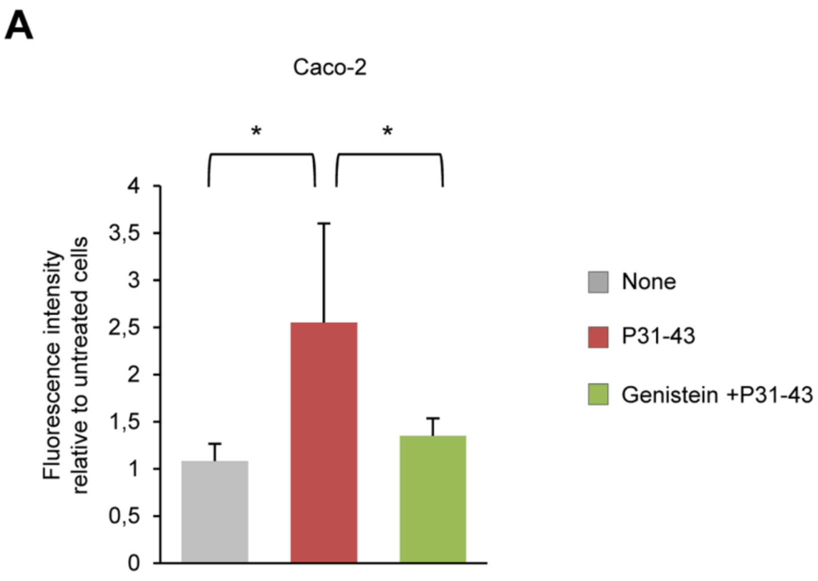

Supplementary Figure 1. Genistein reverses oxidative stress induced by P31-43. The cells were challenged with P31-43 (20µg/ml for 24 hours) in presence or in absence of a 20 min pre-incubation with genistein. Cells were afterwards loaded with CellROX (cellular ROS probe; 5 µM for 10 min at 37°C) and analyzed by confocal microscopy. Quantification of cellular ROS. Means ± SD of three independent experiments. Two paired Student's *t* test, *p<0.05 versus untreated cells and versus P31-43-treated cells.

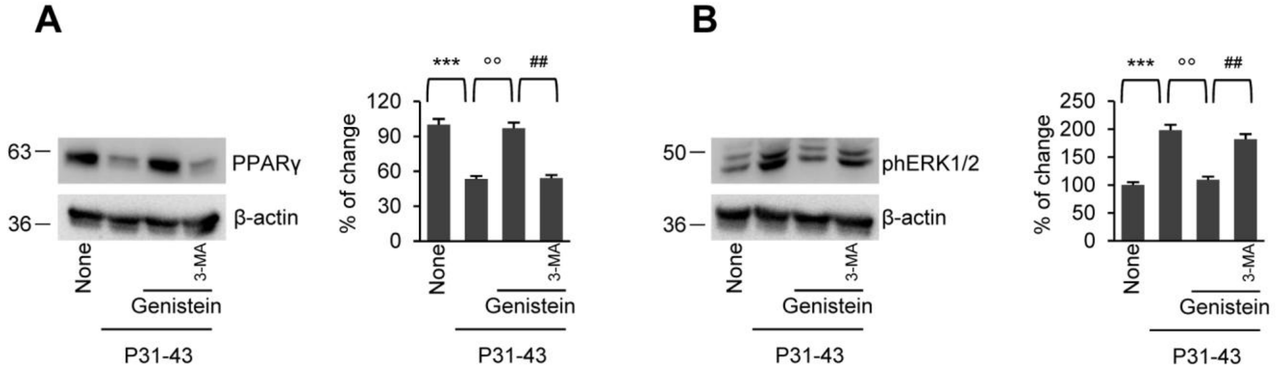

Supplementary Figure 2. 3-MA abrogates the effect of genistein on signs of mucosal inflammation. (**A-B**) Genistein-treated cells were incubated with P31-43 in the presence or absence of 3-MA. Immunoblot of ph-ERK 1/2 (**A**) or PPARγ (**B**) and densitometric analyses of protein levels. β-actin was used as loading control. Means±SD of three independent experiments; ***p<0.001 versus untreated cells, °°p<0.01 versus P31-43-challenged cells and ##p<0.01 versus P31-43-challenged cells pre-treated with Genistein (ANOVA, Bonferroni post-hoc test).
